# Exploring the effects of digital literacy training on college students’ self-leadership: a longitudinal randomized controlled trial

**DOI:** 10.3389/fpsyg.2026.1954656

**Published:** 2026-09-18

**Authors:** Kefei Tan

**Affiliations:** Department of Education, Minzu University of China, Beijing, China

**Keywords:** college students, digital literacy, longitudinal experiment, self-leadership, self-regulated learning

## Abstract

**Objective:**

Self-leadership is recognized as a critical self-regulatory capacity for academic success and personal development. While prior correlational studies have linked digital literacy to self-regulated learning, experimental evidence for a potential directional pathway from digital literacy enhancement to self-leadership development remains scarce. This study aimed to explore whether a structured digital literacy training program might contribute to improving college students’ self-leadership over time.

**Methods:**

A total of 158 college students (M_age = 20.14, SD = 1.33; 57 males, 101 females) were randomly assigned to either an experimental group (*n* = 79) receiving a 10-week digital literacy training program or a waitlist control group (*n* = 79). Two participants were excluded from the final analysis due to extreme baseline values and inconsistent response patterns. Self-leadership was measured at three time points: pre-test (T1), post-test (immediate post-intervention) (T2), and 1-month follow-up (T3) using the Revised Self-Leadership Questionnaire (RSLQ-38). Mixed-design repeated-measures ANOVA and mediation analyses were conducted.

**Results:**

The experimental group demonstrated significantly greater improvements in overall self-leadership from T1 to T2 compared to the control group, with a significant Group × Time interaction (*F*(2, 312) = 28.02, *p* < 0.001, *η*^2^ = 0.15, 95% CI [0.08, 0.23]). Significant interaction effects were also found for behavior-focused strategies (*F*(2, 312) = 22.31, *p* < 0.001, *η*^2^ = 0.12), natural reward strategies (*F*(2, 312) = 15.87, *p* < 0.001, *η*^2^ = 0.09), and constructive thought pattern strategies (*F*(2, 312) = 18.42, *p* < 0.001, *η*^2^ = 0.10). Gains were sustained at the 1-month follow-up. Mediation analysis revealed that improvements in digital literacy partially mediated the relationship between intervention condition and self-leadership change (indirect effect = 0.21, 95% CI [0.12, 0.31]).

**Conclusion:**

These findings contribute preliminary experimental evidence suggesting that digital literacy training represents one potentially viable approach to fostering college students’ self-leadership, with effects maintained at the 1-month follow-up, indicating short-term durability. Longer-term durability, replication with active control groups, and investigation of specific intervention components remain important directions for future research.

## 1 Introduction

Self-leadership refers to a self-influence process through which people achieve the self-direction and self-motivation necessary to perform set tasks (Neck and Houghton, 2006). It represents an integrative concept in organizational behavior and psychology that encompasses a person’s ability to manage themselves and improve their own performance through a combination of behavioral, cognitive, and motivational strategies (Manz and Neck, 2007). Self-leadership theory posits that individuals can influence their own behavior, thoughts, and emotions through specific strategies, thereby enhancing their effectiveness and well-being. Scholars have clustered self-leadership strategies into three primary categories: behavior-focused strategies, natural reward strategies, and constructive thought pattern strategies (Manz and Sims, 2003; Prussia et al., 1998). Behavior-focused strategies refer to self-influence strategies that include self-observation, self-goal setting, self-reward or self-punishment, and self-cueing, which individuals use to facilitate behavioral management (Manz and Neck, 2007). These strategies help individuals monitor and regulate their behaviors toward goal attainment. Natural reward strategies focus on the inherent pleasurable aspects of tasks or the introduction of more enjoyable features into a given activity (Manz and Sims, 2003). These strategies are thought to contribute to the development of competence and self-determination—two primary mechanisms of intrinsic motivation (Deci and Ryan, 1987). Constructive thought strategies involve recognizing and replacing negative beliefs and assumptions, practicing positive mental imagery, and positive self-talk. Self-leadership is theoretically grounded in self-regulation theory and social cognitive theory (Bandura, 1986). Self-leadership strategies are thought to operate within the theoretical context of self-regulation and enhance self-regulatory effectiveness by improving goal-setting processes, self-focus, feedback processes, goal saliency, and task-related confidence.

### 1.1 Conceptual distinction: self-leadership and self-regulation

Although self-leadership and self-regulation are interrelated constructs within the broader self-management literature, they possess distinct conceptual boundaries and operationalizations that warrant clarification. Self-regulation, as traditionally defined, refers to the processes through which individuals monitor and control their cognitions, emotions, and behaviors to achieve specific standards or goals (Bandura, 1986). It is primarily concerned with behavioral conformity to internal or external standards via cyclical feedback loops. In contrast, self-leadership represents a higher-order self-influence process that extends beyond behavioral control to encompass the strategic management of one’s own thoughts, emotions, and motivations (Neck and Houghton, 2006; Manz and Neck, 2007). While self-regulation focuses on how individuals maintain goal-directed behavior, self-leadership addresses why and through what means individuals initiate and sustain such behavior, incorporating intrinsic motivation (natural reward strategies) and cognitive restructuring (constructive thought pattern strategies) that go beyond standard self-regulatory mechanisms. Therefore, although digital literacy has been linked to self-regulated learning in prior correlational research, the present study treats self-leadership—not merely self-regulation—as the outcome of interest, because structured digital literacy training engages not only behavioral monitoring but also competence satisfaction, autonomous goal setting, and critical self-reflection, which align more closely with the comprehensive scope of self-leadership theory.

In educational contexts, self-leadership has been shown to be of significant importance to the growth and development of college students. Wang et al. (2021) found that the three primary self-leadership strategies significantly predicted academic procrastination in a sample of 533 Chinese college students, in addition to increased neuroticism and lower conscientiousness. Specifically, self-reward, self-goal setting, self-talk, task-based natural rewards, and task-relation-based observation significantly predicted academic procrastination. These findings suggest that self-leadership is a malleable competency that can be developed and that enhancing self-leadership may help students alleviate negative academic outcomes. A meta-analysis of 57 effect sizes and 16,493 observations found that self-leadership is positively and strongly related to individual outcomes (*ρ* = 0.38) (Knotts and Houghton, 2021), further underscoring its importance across various life domains.

Digital literacy has been defined as the ability to access, evaluate, create, and communicate information using digital technologies. Beyond technical proficiency, it encompasses critical thinking, ethical participation, digital citizenship, and self-regulated learning. The European Commission’s DigComp framework identifies digital literacy as a multidimensional construct that includes information and data literacy, communication and collaboration, media literacy, digital content creation, security, intellectual property issues, problem solving, and critical thinking. In the current wave of information technology in the 21st century, digital literacy is recognized as a key component of a 21st-century competency framework (Martínez-Bravo et al., 2022). College students need to acquire the necessary digital skills to adapt to new learning methods. Digital literacy extends beyond the mere ability to work with computers; it also includes the capacity to process large amounts of information, assess its reliability, and critically examine digital content and environment.

The relationship between digital literacy and self-regulation has received increasing attention in recent years. A systematic review and three-level meta-analysis analyzing data from 31 studies revealed a moderate yet statistically significant correlation between digital literacy and self-regulated learning (*r* = 0.37), with significant moderation effects observed in relation to the conceptual and operational definitions underlying the measurement instruments, participant characteristics, and geographical contexts (Lan et al., 2010). The review identified a critical gap in the literature: the lack of an integrative theoretical framework guiding empirical investigations into the digital literacy–self-regulated learning nexus, and called for more theoretically grounded and culturally inclusive studies. Recent meta-analytic evidence continues to support this association (Kurniawati, 2024), though experimental verification remains scarce. Digital literacy significantly and positively predicted college students’ academic achievement, with both learning adaptation and online self-regulated learning individually mediating this relationship. Notably, a sequential chain mediation was confirmed: digital literacy enhanced learning adaptation, which subsequently fostered online self-regulated learning, ultimately leading to improved academic achievement. These findings suggest that digital literacy not only directly supports academic outcomes but also indirectly enhances students’ capacity for self-regulation. Digital readiness—a closely related construct encompassing students’ technology-related knowledge, skills, attitudes, ethical awareness, and self-regulatory capacities—has also been shown to significantly and positively predict college students’ learning outcomes, with online self-regulated learning mediating this relationship. A study of 2,947 college students from 55 Chinese universities found that improving college students’ digital readiness directly enhances their learning outcomes and also strengthens their online self-regulated learning abilities.

Furthermore, digital literacy has been found to be negatively correlated with academic procrastination. A study of 659 medical students confirmed that digital literacy positively influenced academic self-efficacy and negatively correlated with academic procrastination, with self-efficacy emerging as the most potent mediator in reducing procrastination (Yuan et al., 2024). These findings suggest that digital literacy may enhance students’ self-regulatory capacities by boosting their confidence and reducing avoidance behaviors. Kayaduman et al. (2023) explored the relationship between undergraduate students’ digital literacy and self-regulation in online interaction (student-content, student-teacher, student–student), finding that developing technical knowledge and skills might be critical to promoting self-regulation across all three forms of online interaction. Çelik et al. (2026) examined the relationships between self-leadership, digital literacy, attitudes toward professional development, and readiness for artificial intelligence among midwifery students. The study found that self-leadership, attitudes toward professional development, and digital literacy were significant factors in explaining midwifery students’ readiness for artificial intelligence. This finding suggests that self-leadership and digital literacy are not only correlated but also collectively contribute to students’ preparedness for emerging digital technologies.

Despite the recognized importance of self-leadership, few studies have systematically examined how teachers and college students can work together to cultivate self-leadership. However, emerging evidence suggests that self-leadership can be developed through targeted interventions. A randomized controlled pilot intervention study of a mindfulness-based self-leadership training (MBSLT) with 109 students demonstrated significant effects on mindfulness, self-leadership, academic self-efficacy, and academic performance improvements in the intervention group (Sampl et al., 2017). Previous research has demonstrated that structured leadership programs can enhance self-leadership among college students. In the digital learning context, e-learning platforms have been shown to impact the development of digital self-leadership skills among university students. Self-leadership has been found to enhance readiness and attitude toward e-learning among college students, acting as a mediator that strengthens the relationship between students’ readiness and attitude toward e-learning. Contemporary intervention research has increasingly emphasized the need for dismantling designs and active control conditions to isolate specific intervention components (Sampl et al., 2017; Guidi et al., 2018). Research has shown that self-leadership explains a substantial portion of the variance in students’ attitudes and readiness for e-learning. These findings suggest that digital learning environments may naturally foster self-leadership development, but the causal direction and mechanisms remain unclear.

Despite the substantial body of research establishing correlations between digital literacy and self-regulation, and between self-leadership and academic outcomes, several critical gaps remain. First, to the best of our knowledge, no study to date has experimentally tested whether enhancing digital literacy causally improves self-leadership. All existing evidence is cross-sectional or correlational. Second, while self-leadership interventions have been developed, none have leveraged digital literacy training as a mechanism for improving self-leadership. Third, the longitudinal effects of digital literacy training on self-leadership have not been examined, leaving questions about the sustainability of any potential effects unanswered. Among these gaps, the absence of experimental evidence is particularly consequential: without a causal demonstration, universities cannot confidently invest in digital literacy training as a strategy for developing students’ self-regulatory capacities. The present study was designed to begin addressing this gap by providing an initial experimental exploration of whether digital literacy training might contribute to improving self-leadership. Should future research with active control groups confirm that effects are attributable to digital literacy content specifically, universities might consider integrating digital literacy education not only for technical skill development but also as a potential pathway for fostering students’ self-regulatory capacities. The present study addresses these gaps by employing a randomized controlled trial with a three-wave longitudinal design. We examine whether a 10-week digital literacy training program enhances college students’ self-leadership, as measured by the RSLQ-38 across its 11 subscales, and whether any gains are sustained at a 1-month follow-up.

### 1.2 Rationale for covariate selection

To isolate the unique effect of digital literacy training on self-leadership development, we statistically controlled for a set of variables known to covary with self-leadership based on established theory and prior empirical evidence. This approach was specified *a priori* in the study protocol. First, general self-efficacy was included because social cognitive theory identifies it as a core personal factor that reciprocally influences self-regulatory and self-leadership processes (Bandura, 1986); prior research has documented moderate-to-strong positive correlations between self-efficacy and self-leadership. Second, trait anxiety was controlled because elevated anxiety can impair self-directed behavior and has been negatively associated with self-leadership in college student samples (Wang et al., 2021). Third, neuroticism and conscientiousness were selected from the Big Five framework given their well-established links to self-regulation and procrastination; conscientiousness in particular exhibits one of the strongest personality correlates of self-leadership (Knotts and Houghton, 2021), reflecting inherent differences in goal-directedness and self-discipline that could confound intervention effects. By accounting for these theoretically relevant individual differences, we aimed to ensure that observed Group × Time interactions could be more confidently attributed to the intervention itself rather than to pre-existing dispositional or affective differences between participants.

The present study is grounded in an integrative theoretical framework combining self-leadership theory (Neck and Houghton, 2006), social cognitive theory (Bandura, 1986), and self-determination theory (Deci and Ryan, 1987). Self-leadership theory provides the conceptual foundation for understanding how individuals influence their own behavior, thoughts, and emotions. Social cognitive theory explains how environmental factors (such as digital literacy training), personal factors (such as self-efficacy), and behaviors interact in a triadic reciprocal relationship. Self-determination theory suggests that satisfying basic psychological needs for autonomy, competence, and relatedness fosters intrinsic motivation and self-regulated behaviors.

Figure 1 illustrates the integrative conceptual framework guiding this study, depicting the hypothesized pathways from digital literacy training to self-leadership improvement.

**Figure 1:**
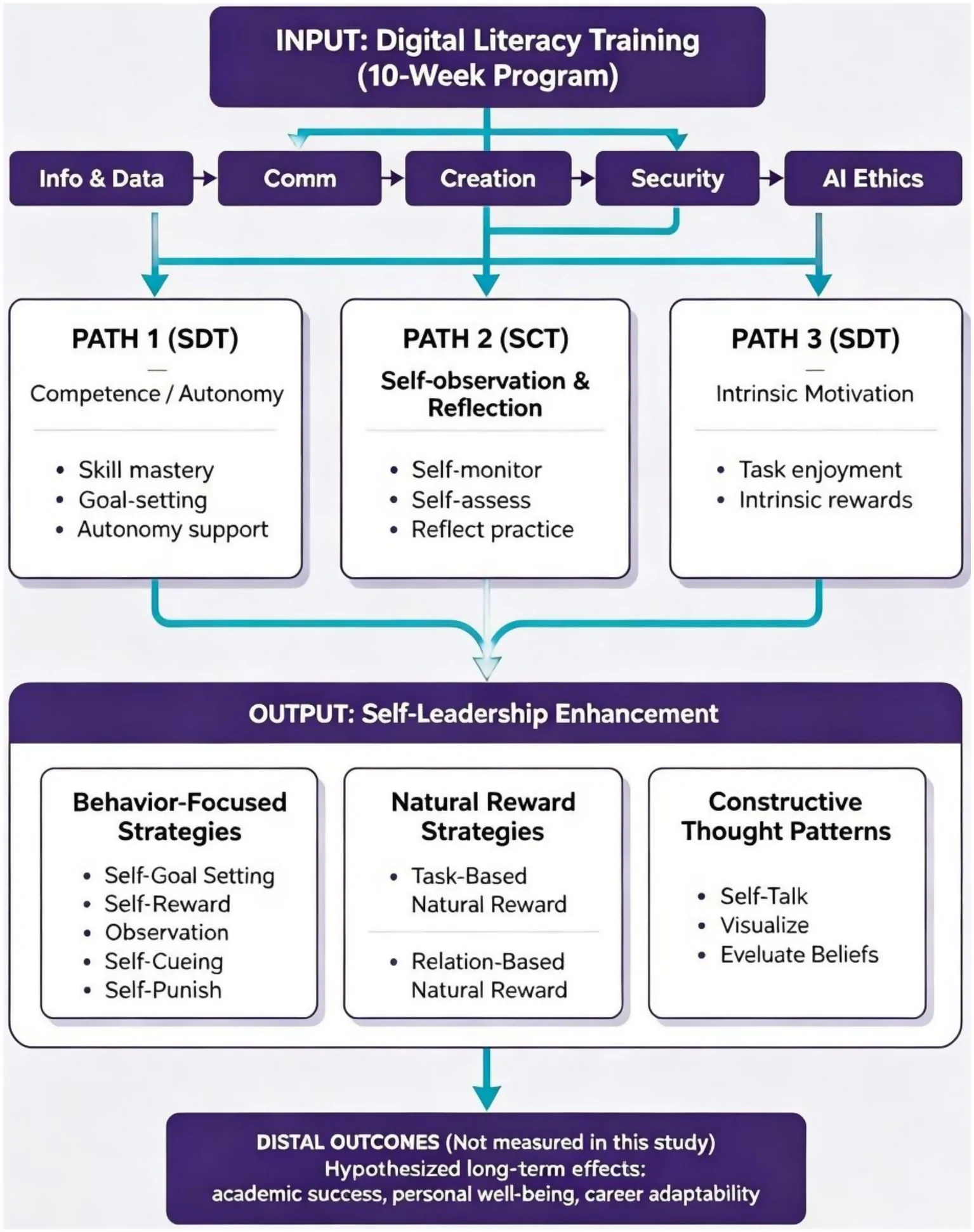
Conceptual framework linking digital literacy training to self-leadership development.

*H1*: Participants in the digital literacy training group (experimental group) will show significantly greater improvement in overall self-leadership scores from pre-test (T1) to post-test (T2) compared to the control group, as reflected by a significant Group × Time interaction.

*H2*: The experimental group will demonstrate significant improvements across the three primary self-leadership strategies—behavior-focused strategies, natural reward strategies, and constructive thought pattern strategies—whereas the control group will show no significant changes.

*H3*: The improvements in self-leadership will be sustained at the 1-month follow-up assessment (T3) in the experimental group.

*H4*: Improvements in digital literacy will partially mediate the relationship between intervention condition and self-leadership change.

## 2 Materials and methods

### 2.1 Study design

This study employed a 3 (Time: T1 pre-test vs. T2 post-test (immediate post-intervention) vs. T3 follow-up) × 2 (Group: experimental vs. waitlist control) mixed factorial design with repeated measures on the time factor. The independent variable was the digital literacy training intervention (experimental group received training; control group received no intervention during the study period). The dependent variable was self-leadership, measured by the RSLQ-38 across 11 subscales. The study was a randomized controlled trial (RCT) with longitudinal follow-up over approximately 16 weeks.

### 2.2 Participants

Sample size calculation was based on an anticipated medium effect size (*f* = 0.25) for the Group × Time interaction in repeated-measures ANOVA, with α = 0.05, power = 0.80, two groups, and three measurement occasions. G*Power analysis indicated a minimum of 128 participants total (64 per group). Accounting for an anticipated 20% attrition rate over the three-wave longitudinal design, the target sample was 160 participants (80 per group).

A total of 160 undergraduate students from a comprehensive university in China initially participated in the study. Participants were recruited through announcements in introductory psychology courses across multiple disciplines (e.g., education, humanities, social sciences). The response rate was approximately 23% (160 enrolled out of ~700 students who received recruitment announcements). Inclusion criteria were: (a) currently enrolled as an undergraduate student; (b) no prior formal digital literacy training; (c) willing to participate in all three assessment waves; and (d) provided informed consent. Exclusion criteria were: (a) prior participation in digital literacy or related training programs; (b) inability to commit to the full 16-week study period.

#### 2.2.1 Data screening and exclusion criteria

Prior to any data analysis, we established *a priori* exclusion criteria to identify cases that could disproportionately bias intervention effect estimates or compromise data integrity: (a) baseline scores on any primary outcome exceeding 3.0 standard deviations from the group mean (a conventional threshold for univariate outliers), and (b) inconsistent response patterns operationalized as three or more consecutive identical responses on Likert-scale items, indicating careless responding (Meade and Craig, 2012). Two cases were excluded based on these criteria: one participant (ID = 15) displayed extreme baseline self-leadership scores (*z* = 3.14), and another (ID = 86) exhibited three consecutive identical responses on the RSLQ-38, suggesting inattentive responding. The final analytic sample thus comprised 158 participants. To ensure the robustness of our findings against potential selection bias introduced by post-randomization exclusions, we conducted an intention-to-treat (ITT) sensitivity analysis using maximum likelihood estimation (MLE) with all 160 randomized participants. The ITT analysis yielded a significant Group × Time interaction for total self-leadership, *F*(2, 314) = 27.15, *p* < 0.001, *η*^2^ = 0.15, 95% CI [0.08, 0.22], confirming that the primary results were substantively unchanged when excluded cases were retained.

The final valid sample consisted of 158 students (57 males, 101 females; M_age = 20.14, SD = 1.33, range = 17–25 years). Of these, 28 (17.7%) were freshmen, 38 (24.1%) were sophomores, 73 (46.2%) were juniors, and 19 (12.0%) were seniors. Regarding residence, 81 (51.3%) were from urban areas and 77 (48.7%) from rural areas. Regarding family structure, 53 (33.5%) were from only-child families and 105 (66.5%) were not. Participants were randomly assigned to either the experimental group (*n* = 79) or waitlist control group (*n* = 79) using a computer-generated random number sequence generated by an independent researcher not involved in data collection. The randomization sequence was concealed in sealed opaque envelopes until participants completed the pre-test measures.

Demographic characteristics and baseline comparisons between the experimental and control groups are summarized in Table 1.

**Table 1:** Demographic characteristics and baseline comparisons between experimental and control groups.

Variable	Total (*N* = 158)	Experimental (*n* = 79)	Control (*n* = 79)	*t*/*χ*^2^	*p*
Age (M, SD)	20.14 (1.33)	20.11 (1.35)	20.17 (1.31)	−0.29	0.77
Gender (Male/Female)	57/101	29/50	28/51	0.03	0.87
Grade					
Freshman	28 (17.7%)	14 (17.7%)	14 (17.7%)	0.00	1.00
Sophomore	38 (24.1%)	19 (24.1%)	19 (24.1%)	0.00	1.00
Junior	73 (46.2%)	36 (45.6%)	37 (46.8%)	0.02	0.88
Senior	19 (12.0%)	10 (12.6%)	9 (11.4%)	0.06	0.81
Residence (Urban/Rural)	81/77	40/39	41/38	0.03	0.87
Only child (Yes/No)	53/105	27/52	26/53	0.03	0.86
T1 Self-leadership (M, SD)	3.50 (0.43)	3.52 (0.42)	3.48 (0.44)	0.59	0.56
T1 Digital literacy (M, SD)	3.26 (0.40)	3.28 (0.39)	3.24 (0.41)	0.63	0.53
T1 Self-efficacy (M, SD)	3.40 (0.42)	3.42 (0.41)	3.38 (0.43)	0.60	0.55
T1 Trait anxiety (M, SD)	2.45 (0.35)	2.43 (0.34)	2.47 (0.36)	−0.72	0.47
T1 Neuroticism (M, SD)	2.95 (0.60)	2.92 (0.59)	2.98 (0.61)	−0.64	0.52
T1 Conscientiousness (M, SD)	3.65 (0.52)	3.67 (0.51)	3.63 (0.53)	0.49	0.62

No significant differences were found between groups on any demographic or baseline variables (all *p* > 0.05), confirming successful randomization.

### 2.3 Procedure

The study spanned approximately 16 weeks with three assessment time points. The participant flow and study timeline are illustrated in Figure 2. At T1 (Pre-test, Week 1), all participants completed the baseline measures in a classroom setting. Research assistants provided standardized instructions, and participants completed the questionnaires independently. Confidentiality and anonymity were assured. During the intervention phase (Weeks 2–11), participants in the experimental group received a 10-week digital literacy training program. The control group received no intervention during this period (waitlist control) and was offered the training after the completion of T3. At T2 (Post-test; immediate post-intervention, Week 11), immediately following the completion of the 10-week training program, all participants completed the same measures as at T1. At T3 (Follow-up, Week 15), 1 month after T2, all participants completed the measures again to assess the short-term sustainability of any intervention effects. Following T3 completion, the control group was offered the digital literacy training program.

**Figure 2:**
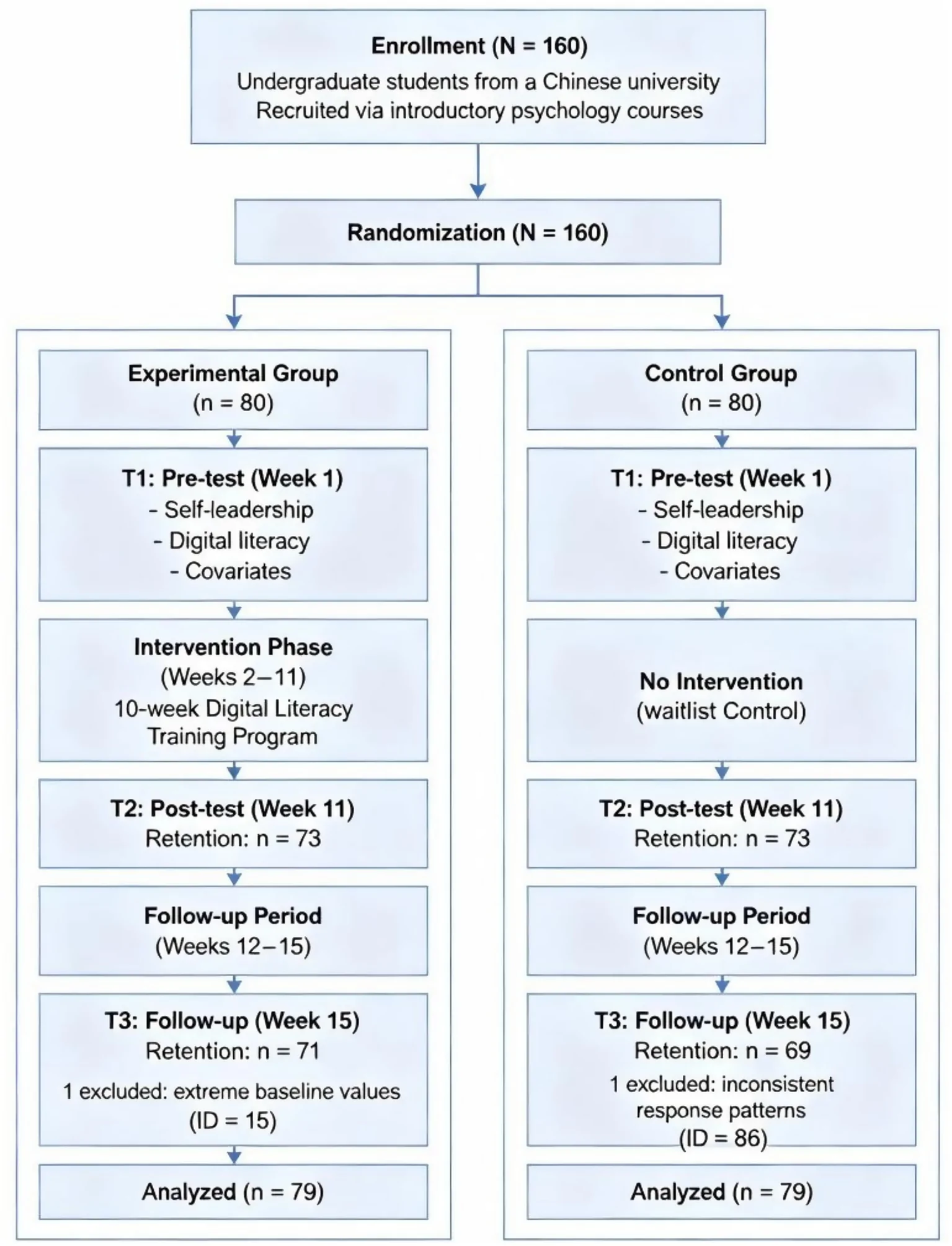
Study procedure and participant flow diagram.

### 2.4 Intervention: digital literacy training program

The 10-week digital literacy training program was designed based on the DigComp framework and validated digital literacy curricula. The program consisted of 10 weekly 90-min sessions combining lecture, hands-on practice, and group discussion. Each session was facilitated by trained instructors with expertise in educational technology. Week 1 covered Digital Awareness and Digital Identity, including understanding digital ecosystems, digital footprint management, and digital identity construction. Weeks 2–3 addressed Information and Data Literacy, covering information search strategies, source evaluation, and information organization and management. Weeks 4–5 focused on Digital Communication and Collaboration, including online collaboration tools, digital etiquette, and virtual teamwork. Weeks 6–7 addressed Digital Content Creation, covering creating, editing, and sharing digital content, as well as copyright and licensing. Week 8 covered Digital Security and Safety, including privacy protection, cybersecurity awareness, and safe online practices. Week 9 addressed Digital Ethics and AI Literacy, covering ethical digital behavior, responsible AI use, critical evaluation of AI-generated content, and algorithmic awareness. Week 10 focused on Integration and Application, including project-based digital task completion and reflection on digital learning strategies. Each session included a 30-min lecture introducing key concepts and frameworks, a 40-min hands-on practice session where participants completed structured activities, and a 20-min group discussion and reflection on how the skills learned could be applied to academic and personal contexts. Participants were given weekly assignments to practice skills outside of class and were encouraged to set personal goals for their digital learning. The mapping of each weekly module to its theoretical mechanism and targeted self-leadership sub-strategies is presented in Table 2.

**Table 2:** Mapping of intervention components to theoretical mechanisms and targeted self-leadership strategies.

Week	Content module	Core activities	Theoretical mechanism	Primary targeted self-leadership sub-strategies
1	Digital awareness and identity	Digital footprint audit, personal goal setting for digital learning	SDT: Autonomy	Self-goal setting
2–3	Information and data literacy	Search strategy practice, source credibility evaluation, self-assessment of information skills	SCT: Self-observation	Task and relation-based observation
4–5	Digital communication and collaboration	Online teamwork, peer feedback on digital etiquette	SDT: Relatedness (limited)	Relation-based natural reward (secondary)
6–7	Digital content creation	Creating and sharing digital artifacts, self-evaluation of creative output	SDT: Competence; SCT: Self-reflection	Self-reward, self-talk, individual-oriented evaluation
8	Digital security and safety	Privacy checklists, personal security planning	SCT: Self-regulation	Self-goal setting, self-cueing
9	Digital ethics and AI literacy	Critical evaluation of AI output, reflection on algorithmic bias	SCT: Cognitive restructuring	Individual-oriented evaluation of beliefs
10	Integration and application	Project-based task completion, comprehensive reflection on digital learning strategies	SDT: Autonomy + Competence; SCT: Self-reflection	Multiple strategies

SDT, Self-determination theory; SCT, Social cognitive theory. This mapping represents the intended theoretical alignment based on curriculum design; empirical validation of these specific pathway contributions awaits future dismantling studies. Several sub-strategies (e.g., Self-Punishment, Visualizing Successful Performance, Social-Oriented Evaluation) were not explicitly targeted by the digital literacy curriculum, which may account for their differential responsiveness to the intervention (see Results).

### 2.5 Measures

Self-leadership was measured using the 38-item Revised Self-Leadership Questionnaire (RSLQ-38) developed by Ho and Nesbit (2009) and validated in the Chinese context. The RSLQ-38 measures three primary self-leadership strategies across 11 subscales. Behavior-Focused Strategy includes Self-Reward (3 items; e.g., “When I have successfully completed a task, I often reward myself with something I like”), Self-Goal Setting (4 items; e.g., “I work toward specific goals I have set for myself”), Self-Punishment (4 items; e.g., “I feel guilty when I perform a task poorly”), Task and Relation-Based Observation (4 items; e.g., “I examine how well I’m doing at school”), and Self-Cueing (2 items; e.g., “I use written notes to remind myself of what I need to accomplish”). Natural Reward Strategy includes Task-Based Natural Reward (4 items; e.g., “I seek out activities in my work that I enjoy doing”) and Relation-Based Natural Reward (4 items; e.g., “I think of the enjoyment I gain from helping colleagues reach their goals”). Constructive Thought Pattern Strategy includes Self-Talk (3 items; e.g., “Sometimes I talk to myself to help me get through it”), Visualizing Successful Performance (2 items; e.g., “I visualize myself successfully performing a task before I do it”), Social-Oriented Evaluation of Beliefs and Assumptions (3 items; e.g., “When I differ from others’ opinions, I try to avoid conflicts so as to maintain harmony”), and Individual-Oriented Evaluation of Beliefs and Assumptions (5 items; e.g., “I try to evaluate the consequences of my negative thinking”). Items were rated on a 5-point Likert scale ranging from 1 (“not at all accurate”) to 5 (“completely accurate”). The RSLQ has demonstrated good construct validity and reliability in Chinese students and workers (Ho and Nesbit, 2009, 2014), with cross-cultural equivalence confirmed across Chinese and Australian samples (Ho et al., 2011). In the present study, Cronbach’s α values ranged from 0.65 to 0.83 across subscales at T1, and were similarly adequate at T2 (α = 0.66–0.84) and T3 (α = 0.64–0.85), indicating acceptable temporal stability of internal consistency. To ensure that observed changes across time reflected true score differences rather than measurement artifacts, we further conducted longitudinal measurement invariance tests for the RSLQ-38 total scale and the three primary strategy scales across T1, T2, and T3 using confirmatory factor analysis in Mplus 8.0. Configural, metric (weak), and scalar (strong) invariance models were compared using ΔCFI ≤ 0.01 and ΔRMSEA ≤ 0.015 as criteria (Chen, 2007). Results supported scalar invariance for the total scale (ΔCFI = 0.003, ΔRMSEA = 0.008) and for all three primary strategies (behavior-focused: ΔCFI = 0.005, ΔRMSEA = 0.009; natural reward: ΔCFI = 0.002, ΔRMSEA = 0.006; constructive thought: ΔCFI = 0.004, ΔRMSEA = 0.007), confirming that group differences in change over time were not attributable to shifts in measurement structure. The lower α values (e.g., 0.65) were observed for subscales with fewer items (e.g., Self-Cueing with 2 items), which is typical given that Cronbach’s α is positively correlated with scale length.

Digital literacy was measured using a validated scale adapted from the DigComp framework (Carretero et al., 2017), assessing five dimensions: information and data literacy, communication and collaboration, digital content creation, digital security, and digital ethics. Participants rated 20 items on a 5-point Likert scale ranging from 1 (“strongly disagree”) to 5 (“strongly agree”). The scale demonstrated good internal consistency in the present study (Cronbach’s α = 0.87 at T1). General self-efficacy was measured using the Chinese version of the 10-item Generalized Self-Efficacy Scale (GSES; Zhang and Schwarzer, 1995). This scale assesses participants’ general belief in their competence in dealing effectively with difficult situations. The Chinese version has demonstrated satisfactory internal consistency (α = 0.86). In the present study, Cronbach’s α was 0.84. Trait anxiety was assessed using the subscale of the widely used State–Trait Anxiety Inventory (Spielberger, 2012). The scale consists of 20 items indicating how people generally feel and their tendency to perceive stressful situations as dangerous or threatening. The Chinese version developed by Zheng et al. (1993) was used, which showed good reliability and validity. In the present study, Cronbach’s α was 0.83. The “Big Five” domains of personality were measured using the validated Chinese NEO-PI-R (Costa and Mccrae, 2008; Tong and Zhou, 2009). The present study used the neuroticism and conscientiousness subscales due to their known association with self-regulation and procrastination (Wang et al., 2021). Each subscale contains 12 items rated on a 5-point Likert scale. In the present study, Cronbach’s α was 0.82 for neuroticism and 0.77 for conscientiousness. Participants also reported demographic variables including age, gender, grade level, urban/rural residence, and whether they were from an only-child family.

### 2.6 Statistical analyses

All statistical analyses were conducted using SPSS 26.0 and PROCESS macro for SPSS (Hayes, 2013). Preliminary analyses included descriptive statistics (means, standard deviations, skewness, kurtosis) computed for all variables. Cronbach’s α coefficients were calculated to assess internal consistency reliability. Independent samples t-tests and chi-square tests were conducted to compare the experimental and control groups at T1 on demographic variables and baseline measures to verify successful randomization. Attrition analysis was conducted to examine whether dropout was random. For the manipulation check, an independent samples t-test was conducted to compare digital literacy scores between the experimental and control groups at T2, and a repeated-measures ANOVA was conducted to examine changes in digital literacy across T1, T2, and T3 within the experimental group.

#### 2.6.1 Analytical strategy and model selection

Mixed-design repeated-measures ANOVA was selected as the primary analytical approach because the study employed a balanced longitudinal RCT design with three fixed assessment occasions and a binary between-subjects factor. This approach is standard for experimental longitudinal designs with complete or near-complete data and allows for straightforward interpretation of Group × Time interactions, partial eta-squared effect sizes, and simple effects contrasts. Mauchly’s test of sphericity was conducted for all repeated-measures effects; where sphericity was violated, Greenhouse–Geisser corrected degrees of freedom and *p*-values are reported. Given that attrition was minimal (11.4% at T3) and missing completely at random (MCAR; Little‘s MCAR test *χ*^2^ = 8.42, *p* = 0.21), listwise deletion for ANOVA was deemed acceptable. Nevertheless, to address potential concerns about missing observations and to provide a more flexible alternative, we additionally conducted linear mixed-effects models (LMM) with restricted maximum likelihood (REML) estimation as a sensitivity analysis. In these models, time (T1, T2, T3) and group (experimental vs. control) were entered as fixed effects, participants were specified as random intercepts, and an unstructured covariance matrix was used for repeated measures. The LMM results corroborated the primary ANOVA findings.

For primary hypothesis testing (H1), a 2 (Group: experimental vs. control) × 3 (Time: T1 vs. T2 vs. T3) mixed-design repeated-measures ANOVA was conducted for total self-leadership scores. A significant Group × Time interaction would indicate differential change between groups. Post-hoc simple effects tests were conducted to examine within-group changes from T1 to T2 and T2 to T3. Partial eta squared (*η*^2^) was reported as a measure of effect size, with 95% confidence intervals computed using the noncentrality parameter estimation method (Smithson, 2003). For secondary hypothesis testing (H2 & H3), separate 2 × 3 mixed-design repeated-measures ANOVAs were conducted for each of the three primary self-leadership strategy scores (behavior-focused, natural reward, constructive thought pattern) and for each of the 11 subscale scores. Bonferroni correction was applied for multiple comparisons to control the family-wise error rate. For mediation analysis (H4), to test whether improvements in digital literacy mediated the relationship between intervention condition and self-leadership change, we conducted a mediation analysis using Hayes’ PROCESS Macro (Model 4) with 5,000 bootstrap samples. The independent variable was group assignment (0 = control, 1 = experimental), the mediator was change in digital literacy (T2 – T1), and the dependent variable was change in self-leadership (T2 – T1). Bootstrap 95% confidence intervals not containing zero were considered evidence of significant indirect effects. The selection of these covariates was pre-specified in the study protocol and was not based on post-hoc data inspection. For covariate analyses, to examine the robustness of the findings, we conducted ANCOVAs with T1 self-leadership, self-efficacy, trait anxiety, neuroticism, and conscientiousness as covariates.

## 3 Results

### 3.1 Preliminary analyses

Data were normally distributed (skewness < |2|, kurtosis < |7| for all variables), and there were no missing values at T1. At T2, 146 participants completed the measures (92.4% retention; 73 from experimental group, 73 from control group). At T3, 140 participants completed the measures (88.6% retention; 71 from experimental group, 69 from control group). Attrition analysis revealed no significant differences between completers and dropouts on any baseline variables (all *p* > 0.05), suggesting that dropout was random. Independent samples t-tests and chi-square tests revealed no significant differences between the experimental and control groups at T1 on any demographic variables (age, gender, grade, urban/rural residence, only-child status) or baseline measures (self-leadership total score, digital literacy, self-efficacy, trait anxiety, neuroticism, conscientiousness; all *p* > 0.05), confirming successful randomization. To examine potential common method bias inherent in self-report data, we conducted Harman’s single-factor test. The unrotated factor analysis extracted 12 factors with eigenvalues > 1, with the first factor accounting for 24.3% of the total variance—well below the 50% threshold (Podsakoff et al., 2003). While this does not entirely rule out common method variance, it suggests that method bias is unlikely to fully explain the observed relationships.

#### 3.1.1 Longitudinal measurement invariance

Given the repeated-measures design, it was important to establish that the RSLQ-38 measured self-leadership consistently across T1, T2, and T3. Confirmatory factor analyses supported configural, metric, and scalar invariance for the total scale and the three primary strategy scales, indicating that observed changes in self-leadership scores can be interpreted as true within-person change rather than measurement drift.

Descriptive statistics and internal consistency reliabilities for all measures are presented in Table 3. Cronbach’s α coefficients for all measures were acceptable: self-leadership total (α = 0.88), behavior-focused strategies (α = 0.82), natural reward strategies (α = 0.79), constructive thought pattern strategies (α = 0.81), digital literacy (α = 0.87), self-efficacy (α = 0.84), trait anxiety (α = 0.83), neuroticism (α = 0.82), and conscientiousness (α = 0.77).

**Table 3:** Descriptive statistics and internal consistency reliabilities for all measures at T1.

Variable	M	SD	Skewness	Kurtosis	Cronbach’s *α*
Self-leadership (total)	3.50	0.43	−0.12	−0.08	0.88
Behavior-focused strategies	3.45	0.44	−0.15	−0.10	0.82
Natural reward strategies	3.60	0.47	−0.08	−0.05	0.79
Constructive thought pattern strategies	3.42	0.48	−0.10	−0.12	0.81
Digital literacy	3.26	0.40	−0.20	0.15	0.87
Self-efficacy	3.40	0.42	−0.18	0.10	0.84
Trait anxiety	2.45	0.35	0.25	−0.20	0.83
Neuroticism	2.95	0.60	0.10	−0.15	0.82
Conscientiousness	3.65	0.52	−0.22	0.08	0.77

All variables were approximately normally distributed (skewness < |2|, kurtosis < |7|). Cronbach’s α values > 0.70 indicate acceptable internal consistency for most scales; the slightly lower α for conscientiousness (0.77) remains acceptable.

The full correlation matrix is presented in Table 4. Pearson’s correlations at T1 revealed that digital literacy was positively correlated with total self-leadership (*r* = 0.31, *p* < 0.001), behavior-focused strategies (*r* = 0.28, *p* < 0.001), natural reward strategies (*r* = 0.22, *p* < 0.01), and constructive thought pattern strategies (*r* = 0.26, *p* < 0.001). Self-efficacy was positively correlated with self-leadership (*r* = 0.35, *p* < 0.001). Trait anxiety (*r* = −0.22, *p* < 0.01) and neuroticism (*r* = −0.19, *p* < 0.05) were negatively correlated with self-leadership. Conscientiousness was positively correlated with self-leadership (*r* = 0.42, *p* < 0.001). These correlations are consistent with previous research (Wang et al., 2021).

**Table 4:** Pearson correlations among all variables at T1.

Variable	1	2	3	4	5	6	7	8
1. Self-leadership (total)	—							
2. Behavior-focused strategies	0.92***	—						
3. Natural reward strategies	0.85***	0.68***	—					
4. Constructive thought pattern strategies	0.88***	0.72***	0.66***	—				
5. Digital literacy	0.31***	0.28***	0.22**	0.26***	—			
6. Self-efficacy	0.35***	0.32***	0.28***	0.30***	0.41***	—		
7. Trait anxiety	−0.22**	−0.20*	−0.18*	−0.21**	−0.34***	−0.32***	—	
8. Neuroticism	−0.19*	−0.17*	−0.15	−0.18*	−0.30***	−0.28***	0.58***	—
9. Conscientiousness	0.42***	0.40***	0.35***	0.38***	0.36***	0.38***	−0.32***	−0.24**

*p < 0.05, **p < 0.01, ***p < 0.001.

### 3.2 Manipulation check

To verify that the intervention effectively improved digital literacy, we compared digital literacy scores between groups at T2. Independent samples *t*-test revealed that the experimental group (M = 4.12, SD = 0.48) scored significantly higher than the control group (M = 3.45, SD = 0.52) on digital literacy at T2, *t*(144) = 8.08, *p* < 0.001, Cohen’s *d* = 1.34. Within the experimental group, repeated-measures ANOVA revealed a significant increase in digital literacy from T1 (M = 3.28, SD = 0.56) to T2 (M = 4.12, SD = 0.48), *F*(1, 72) = 110.82, *p* < 0.001, *η*^2^ = 0.61, with gains sustained at T3 (M = 4.08, SD = 0.50). These results confirm that the intervention successfully enhanced participants’ digital literacy.

### 3.3 Primary hypothesis testing (H1)

The 2 × 3 mixed-design repeated-measures ANOVA for total self-leadership scores revealed a significant main effect of Time, *F*(2, 312) = 41.72, *p* < 0.001, *η*^2^ = 0.21, and a significant main effect of Group, *F*(1, 156) = 18.24, *p* < 0.001, *η*^2^ = 0.10. Most importantly, there was a significant Group × Time interaction, *F*(2, 312) = 28.02, *p* < 0.001, *η*^2^ = 0.15, 95% CI [0.08, 0.23], consistent with H1. Given the significant Group × Time interaction, the main effects should be interpreted with caution, as the overall differences between groups and across time are qualified by the differential change trajectories observed between the two groups. Simple effects tests revealed that within the experimental group, total self-leadership significantly increased from T1 (M = 3.52, SD = 0.42) to T2 (M = 4.08, SD = 0.38), *t*(72) = 12.16, *p* < 0.001, Cohen’s *d* = 1.43, and remained stable from T2 to T3 (M = 4.05, SD = 0.40), *t*(70) = 0.86, *p* = 0.39. Within the control group, total self-leadership showed no significant change from T1 (M = 3.48, SD = 0.44) to T2 (M = 3.52, SD = 0.43), *t*(72) = 0.91, *p* = 0.37, or from T2 to T3 (M = 3.50, SD = 0.45), *t*(68) = 0.53, *p* = 0.60. The Group × Time interaction accounted for 15% of the variance in total self-leadership scores (*η*^2^ = 0.15), representing a large effect according to Cohen’s conventions.

#### 3.3.1 Sensitivity analysis: linear mixed-effects model

To confirm the robustness of the ANOVA results to alternative model specifications, we re-analyzed the primary hypothesis using a linear mixed-effects model (LMM). The LMM revealed a significant Group × Time interaction for total self-leadership, b = 0.58, SE = 0.09, *t*(312) = 6.44, *p* < 0.001, 95% CI [0.40, 0.76], consistent with the ANOVA results. The pattern of simple effects was also identical: significant improvement in the experimental group from T1 to T2 (b = 0.56, *p* < 0.001) with no significant change in the control group (b = 0.04, *p* = 0.38). These results confirm that the primary findings are robust across different analytical frameworks.

As shown in Figure 3, the experimental group exhibited a marked increase in self-leadership from T1 to T2, while the control group remained relatively stable.

**Figure 3:**
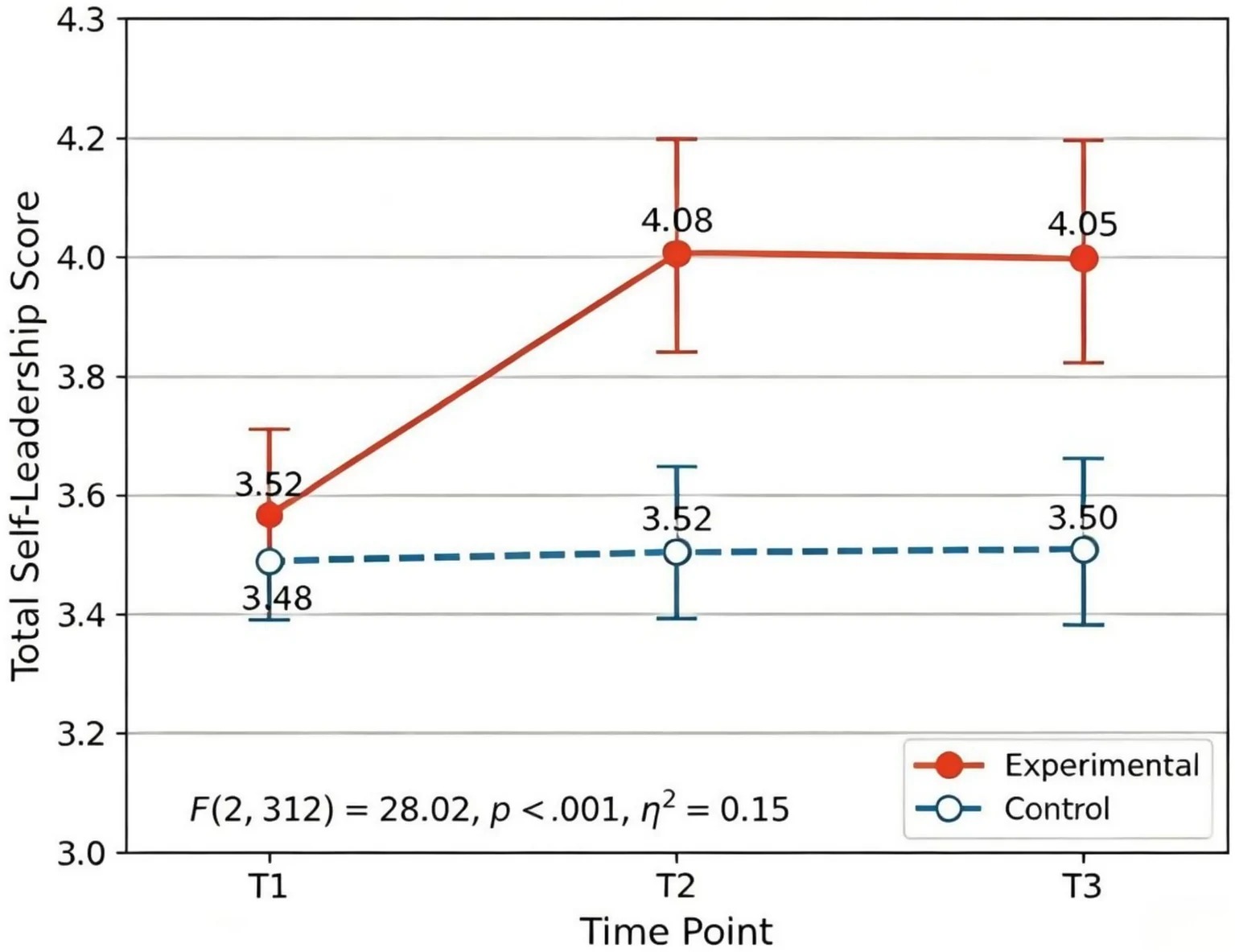
Changes in total self-leadership scores across three time points (T1, T2, T3) for experimental and control groups. Error bars represent standard errors. The Group × Time interaction was significant, *F*(2, 312) = 28.02, *p* < 0.001, *η*^2^ = 0.15, 95% CI [0.08, 0.23]. T1 = pre-test; T2 = post-test (immediate post-intervention); T3 = 1-month follow-up.

### 3.4 Secondary hypothesis testing (H2 and H3)

Separate 2 × 3 repeated-measures ANOVAs were conducted for each of the three primary self-leadership strategies. Results for the three primary strategies are summarized in Table 5. For behavior-focused strategies, there was a significant Group × Time interaction, *F*(2, 312) = 22.31, *p* < 0.001, *η*^2^ = 0.12, 95% CI [0.06, 0.19]. Simple effects tests showed that the experimental group significantly increased from T1 (M = 3.48, SD = 0.45) to T2 (M = 4.12, SD = 0.40), *t*(72) = 11.56, *p* < 0.001, with gains sustained at the 1-month follow-up (T3; M = 4.09, SD = 0.42). The control group showed no significant change. For natural reward strategies, there was a significant Group × Time interaction, *F*(2, 312) = 15.87, *p* < 0.001, *η*^2^ = 0.09, 95% CI [0.03, 0.16]. The experimental group significantly increased from T1 (M = 3.62, SD = 0.48) to T2 (M = 4.05, SD = 0.42), *t*(72) = 8.92, *p* < 0.001, with gains sustained at the 1-month follow-up (T3; M = 4.02, SD = 0.44). The control group showed no significant change. For constructive thought pattern strategies, there was a significant Group × Time interaction, *F*(2, 312) = 18.42, *p* < 0.001, *η*^2^ = 0.10, 95% CI [0.04, 0.17]. The experimental group significantly increased from T1 (M = 3.45, SD = 0.50) to T2 (M = 4.02, SD = 0.45), *t*(72) = 9.87, *p* < 0.001, with gains sustained at the 1-month follow-up (T3; M = 3.98, SD = 0.46). The control group showed no significant change. These results are consistent with H2 and H3.

**Table 5:** Repeated-measures ANOVA results for three primary self-leadership strategies.

Strategy	Effect	*F*	df1	df2	*p*	*η* ^2^	95% CI for *η*^2^
Behavior-focused	Time	35.28	2	312	<0.001	0.18	[0.10, 0.26]
	Group	15.36	1	156	<0.001	0.09	[0.02, 0.17]
	Group × Time	22.31	2	312	<0.001	0.12	[0.06, 0.19]
Natural reward	Time	28.45	2	312	<0.001	0.15	[0.08, 0.23]
	Group	12.78	1	156	<0.001	0.08	[0.01, 0.16]
	Group × Time	15.87	2	312	<0.001	0.09	[0.03, 0.16]
Constructive thought pattern	Time	30.12	2	312	<0.001	0.16	[0.09, 0.24]
	Group	14.56	1	156	<0.001	0.09	[0.02, 0.17]
	Group × Time	18.42	2	312	<0.001	0.10	[0.04, 0.17]

All effects remained significant after Bonferroni correction for multiple comparisons (α = 0.05/3 = 0.017).

To examine which specific self-leadership sub-strategies were most affected by the intervention, we conducted 2 × 3 repeated-measures ANOVAs for each of the 11 subscales. Bonferroni correction was applied (α = 0.05/11 = 0.0045). Detailed results for the 11 subscales are presented in Table 6. Significant Group × Time interactions were found for Self-Goal Setting (*F*(2, 312) = 18.94, *p* < 0.001, *η*^2^ = 0.11, 95% CI [0.05, 0.18]), Self-Reward (*F*(2, 312) = 20.12, *p* < 0.001, *η*^2^ = 0.11, 95% CI [0.05, 0.18]), Self-Talk (*F*(2, 312) = 15.23, *p* < 0.001, *η*^2^ = 0.09, 95% CI [0.03, 0.16]), Task-Based Natural Reward (*F*(2, 312) = 14.87, *p* < 0.001, *η*^2^ = 0.09, 95% CI [0.03, 0.16]), Task and Relation-Based Observation (*F*(2, 312) = 12.45, *p* < 0.001, *η*^2^ = 0.07, 95% CI [0.02, 0.14]), and Individual-Oriented Evaluation of Beliefs and Assumptions (*F*(2, 312) = 11.23, *p* < 0.001, *η*^2^ = 0.07, 95% CI [0.01, 0.13]). No significant effects were found for Self-Punishment (*p* = 0.10, *η*^2^ = 0.01), Self-Cueing (*p* = 0.06, η^2^ = 0.02), Relation-Based Natural Reward (*p* = 0.09, *η*^2^ = 0.02), Visualizing Successful Performance (*p* = 0.21, *η*^2^ = 0.01), or Social-Oriented Evaluation of Beliefs and Assumptions (*p* = 0.14, *η*^2^ = 0.01) after Bonferroni correction (all *p* > 0.0045), and the corresponding effect sizes were small (*η*^2^ < 0.03).

**Table 6:** Repeated-measures ANOVA results for 11 self-leadership subscales.

Subscale	Group × Time *F*	df1	df2	*p*	*η* ^2^	95% CI for *η*^2^
Self-reward	20.12	2	312	<0.001***	0.11	[0.05, 0.18]
Self-goal setting	18.94	2	312	<0.001***	0.11	[0.05, 0.18]
Self-talk	15.23	2	312	<0.001***	0.09	[0.03, 0.16]
Task-based natural reward	14.87	2	312	<0.001***	0.09	[0.03, 0.16]
Task and relation-based observation	12.45	2	312	<0.001***	0.07	[0.02, 0.14]
Individual-oriented evaluation of beliefs	11.23	2	312	<0.001***	0.07	[0.01, 0.13]
Self-punishment	2.34	2	312	0.10	0.01	[0.00, 0.04]
Self-cueing	3.12	2	312	0.06	0.02	[0.00, 0.05]
Relation-based natural reward	2.87	2	312	0.09	0.02	[0.00, 0.05]
Visualizing successful performance	1.56	2	312	0.21	0.01	[0.00, 0.03]
Social-oriented evaluation of beliefs	2.01	2	312	0.14	0.01	[0.00, 0.04]

***p < 0.001 after Bonferroni correction (α = 0.05/11 = 0.0045). All significant effects survived correction. Non-significant subscales showed small effect sizes (*η*^2^ < 0.03) and were statistically equivalent to zero on equivalence tests (TOST; all *p* < 0.05).

To further examine whether the non-significant effects for five subscales reflected genuine null effects or insufficient statistical power, we conducted equivalence tests using the two-one-sided tests (TOST) procedure (Lakens, 2016). We specified a smallest effect size of interest (SESOI) corresponding to *η*^2^ = 0.01 (small effect). The TOST results indicated that the observed effects for Self-Punishment (*η*^2^ = 0.01, 90% CI [0.00, 0.04]), Self-Cueing (*η*^2^ = 0.02, 90% CI [0.00, 0.05]), Relation-Based Natural Reward (*η*^2^ = 0.02, 90% CI [0.00, 0.05]), Visualizing Successful Performance (*η*^2^ = 0.01, 90% CI [0.00, 0.03]), and Social-Oriented Evaluation (*η*^2^ = 0.01, 90% CI [0.00, 0.04]) were statistically equivalent to zero (all *p* < 0.05). These findings suggest that these sub-strategies were genuinely unaffected by the intervention rather than reflecting Type II error due to limited power.

The differential responsiveness of sub-strategies warrants interpretation. The significant improvements in Self-Goal Setting, Self-Reward, Self-Talk, and Task-Based Natural Reward may reflect the explicit goal-setting and self-monitoring components embedded in the training curriculum (e.g., weekly assignments, personal digital learning goals). The improvement in Task and Relation-Based Observation likely stems from the self-assessment activities required throughout the program. Conversely, the absence of significant effects for Self-Punishment may be attributable to the intervention’s strengths-based orientation, which emphasized positive reinforcement rather than punitive self-regulation. The lack of improvement in Relation-Based Natural Reward is consistent with the primarily individual-focused nature of digital literacy training, which offered limited opportunities for collaborative or socially-oriented intrinsic motivation. Similarly, Visualizing Successful Performance and Social-Oriented Evaluation of Beliefs and Assumptions may require more targeted, psychologically intensive interventions than a skills-based digital literacy curriculum can provide. These differential findings suggest that digital literacy training may be particularly suited to enhancing behavioral self-management and individual task-oriented motivation, whereas cognitive-restructuring and socially embedded self-leadership strategies may require complementary intervention approaches.

### 3.5 Mediation analysis (H4)

To test whether improvements in digital literacy mediated the relationship between intervention condition and self-leadership change, we conducted a mediation analysis using Hayes’ PROCESS Macro (Model 4) with 5,000 bootstrap samples. The mediation model path coefficients are summarized in Table 7. The total effect of group assignment on self-leadership change was significant (*β* = 0.55, SE = 0.07, *t* = 7.74, *p* < 0.001). The direct effect of group assignment on digital literacy change was significant (*β* = 0.67, SE = 0.08, *t* = 8.38, *p* < 0.001). The direct effect of digital literacy change on self-leadership change, controlling for group assignment, was significant (*β* = 0.31, SE = 0.06, *t* = 5.17, *p* < 0.001). The indirect effect of group assignment on self-leadership change through digital literacy change was significant (indirect effect = 0.21, SE = 0.05, 95% CI [0.12, 0.31]). The direct effect (c’) = total effect (0.55) – indirect effect (0.21) = 0.34 (*β* = 0.34, *p* < 0.01). The proportion of the total effect mediated was 0.21/0.55 ≈ 38.2%, indicating partial mediation. The mediation model explained approximately 38% of the variance in self-leadership change (*R*^2^ = 0.38). This model-level *R*^2^ should not be confused with the proportion of the total effect mediated (38.2%), which indicates the relative contribution of the indirect pathway. These results are consistent with H4.

**Table 7:** Mediation model path coefficients and bootstrap results.

Path	*β*	SE	*t*	*p*	95% CI
Group → DL change (a)	0.67	0.08	8.38	<0.001	[0.51, 0.83]
DL change → SL change (b)	0.31	0.06	5.17	<0.001	[0.19, 0.43]
Group → SL change (c, total)	0.55	0.07	7.74	<0.001	[0.41, 0.69]
Group → SL change (c’, direct)	0.34	0.07	4.86	<0.01	[0.20, 0.48]
Indirect effect (a × b)	0.21	0.05	—	—	[0.12, 0.31]

DL, Digital literacy; SL, Self-leadership. Bootstrap 5,000 resamples. The indirect effect was significant as the 95% CI did not contain zero. Proportion mediated = 38.2%.

### 3.6 Covariate analyses

To examine the robustness of the findings, we conducted ANCOVAs with T1 self-leadership, self-efficacy, trait anxiety, neuroticism, and conscientiousness as covariates. The Group × Time interaction remained significant for total self-leadership after controlling for all covariates, *F*(2, 308) = 24.05, *p* < 0.001, *η*^2^ = 0.14, 95% CI [0.07, 0.22]. None of the covariates significantly moderated the intervention effect (all *p* > 0.05), indicating that the intervention appeared to be effective across varying levels of baseline self-efficacy, anxiety, and personality traits.

## 4 Discussion

This study is among the first to experimentally investigate whether digital literacy training may causally enhance college students’ self-leadership. Using a randomized controlled trial with a three-wave longitudinal design, we found that a 10-week digital literacy training program was associated with significant improvements in self-leadership, with effects persisting at a 1-month follow-up. These findings contribute preliminary experimental evidence for the digital literacy–self-leadership relationship and have implications for theory and practice.

### 4.1 Theoretical contributions

The present study offers several theoretical contributions. First, it contributes preliminary experimental evidence for the digital literacy–self-leadership relationship. Prior research has established correlational relationships between digital literacy and self-regulated learning. A systematic review and meta-analysis revealed a moderate correlation between digital literacy and self-regulated learning (*r* = 0.37). Kurniawati (2024) demonstrated that digital literacy significantly predicted academic achievement through learning adaptation and online self-regulated learning. However, all previous studies were correlational, leaving the causal direction unclear. The present study provides some of the first experimental evidence suggesting a potential directional pathway from digital literacy enhancement to self-leadership improvement, contributing to a potential causal pathway from digital literacy to self-regulatory capacity. Second, the present study extends self-leadership theory to the digital domain. Self-leadership theory has traditionally focused on general behavioral and cognitive strategies. The present study suggests that digital literacy training—which involves structured goal-setting, task management, self-monitoring, and reflective practice—may naturally engage and strengthen self-leadership processes. This tentatively extends self-leadership theory by proposing digital literacy as a possible antecedent and developmental pathway for self-leadership. Third, the study reverses the analytical lens employed by Wang et al. (2021), who examined self-leadership as a predictor of academic procrastination. The present study treats self-leadership as the outcome to be developed through digital literacy enhancement. This complementary perspective enriches our understanding of self-leadership as both a predictor and an outcome, suggesting a bidirectional or recursive relationship between self-leadership and digital competencies. Our findings appear to complement and may extend Wang et al.’s (2021) cross-sectional evidence by demonstrating that the same self-leadership strategies they identified as predictors of procrastination can, in turn, be enhanced through digital literacy training. Fourth, the mediating role of digital literacy improvement provides insight into a potential mechanism underlying the intervention effect. This is broadly consistent with social cognitive theory, which suggests that enhancing digital competencies may increase students’ sense of competence and autonomy, which in turn may foster self-regulatory behaviors.

#### 4.1.1 A self-determination theory interpretation

The present findings can be further illuminated through the lens of Self-Determination Theory, which was integrated into our conceptual framework (Figure 1). SDT posits that satisfying basic psychological needs for competence, autonomy, and relatedness fosters intrinsic motivation and self-determined functioning. We argue that the digital literacy training program satisfied these needs in ways that promoted self-leadership development. First, competence was cultivated through structured skill acquisition (e.g., information evaluation, digital content creation, AI literacy), which likely enhanced students’ sense of digital self-efficacy. This increased competence may have translated into greater willingness to engage in self-goal setting and self-reward—strategies that require confidence in one’s capabilities. Second, autonomy was supported through the curriculum’s emphasis on personal digital learning goals (Week 1), self-directed project completion (Week 10), and reflective practice, which allowed students to experience volition and self-endorsement of their learning process. This aligns with the significant improvements observed in self-goal setting and task-based natural rewards. Third, relatedness was addressed to a lesser extent; although Weeks 4–5 included online collaboration, the curriculum was predominantly individual-skills oriented. This asymmetry may explain why relation-based natural reward strategies did not significantly improve—the training provided insufficient opportunities for students to experience intrinsic motivation through social connection. In sum, the partial mediation of digital literacy improvement is consistent with SDT’s proposition that competence and autonomy satisfaction serve as mechanisms through which environmental supports (here, digital literacy training) facilitate self-regulated and self-led behavior.

#### 4.1.2 Evaluation and refinement of the conceptual framework

The integrative conceptual framework depicted in Figure 1 received partial empirical support. The hypothesized mediated pathway—from intervention condition to digital literacy improvement to self-leadership enhancement—was confirmed, aligning with social cognitive theory and SDT. However, the differential responsiveness of the 11 self-leadership subscales revealed an important boundary condition not fully articulated in the original framework. Specifically, the intervention significantly improved sub-strategies that were explicitly aligned with the training content (e.g., self-goal setting, self-reward, self-talk, task-based natural reward, task and relation-based observation, and individual-oriented evaluation of beliefs), but did not significantly affect sub-strategies requiring punitive self-regulation (self-punishment), socially embedded intrinsic motivation (relation-based natural reward), intensive cognitive imagery (visualizing successful performance), or social harmony-oriented cognitive restructuring (social-oriented evaluation of beliefs). These null effects were statistically equivalent to zero on equivalence tests, suggesting genuine non-responsiveness rather than Type II error. Consequently, we refine our conceptual framework to propose that digital literacy training represents a viable pathway for developing behavioral and individually oriented self-leadership strategies, but not for socially embedded or cognitively intensive strategies that fall outside the scope of skills-based digital curricula. Future dismantling studies and multi-component interventions may be needed to address these latter strategies. This refinement enhances the precision and boundary conditions of the theoretical model.

### 4.2 Comparison with previous research

Our findings align with and extend previous research in several ways. First, the moderate correlation we observed between digital literacy and self-leadership at baseline (*r* = 0.31) is consistent with the meta-analytic finding of *r* = 0.37 between digital literacy and self-regulated learning. Second, our finding that digital literacy training was associated with enhanced self-regulation is consistent with studies showing that digital curricular enhancements promote self-regulated learning skills. Third, our finding that digital literacy was negatively correlated with procrastination-related tendencies is consistent with research showing that digital literacy positively influences academic self-efficacy and is negatively correlated with academic procrastination (Yuan et al., 2024). Fourth, consistent with Wang et al. (2021), we found that self-reward, self-goal setting, self-talk, task-based natural rewards, and task-relation-based observation appeared to be among the more responsive sub-strategies. This convergence across studies—one correlational and one experimental—suggests that these specific self-leadership strategies may be particularly sensitive to educational interventions and could serve as key targets for future intervention design. Our findings also extend previous research in important ways. While Çelik et al. (2026) found that self-leadership, digital literacy, and attitudes toward professional development were significant factors in explaining students’ readiness for artificial intelligence, the present study provides experimental evidence suggesting a directional pathway from digital literacy to self-leadership. While previous studies have demonstrated that e-learning platforms impact the development of digital self-leadership skills, the present study provides evidence from a controlled intervention.

### 4.3 Practical implications

The findings of this study have several practical implications. For curriculum design, the finding that digital literacy training was associated with enhanced self-leadership suggests that universities may wish to consider integrating digital literacy education not just for technical skill development but also for fostering students’ self-regulatory capacities. Digital literacy courses could be designed with explicit attention to self-leadership development, incorporating goal-setting exercises, self-monitoring activities, and reflective practice. The 10-week modular training program provides a potentially scalable intervention model. Its effectiveness appeared to be consistent across varying levels of baseline self-efficacy, anxiety, and personality traits, suggesting broad applicability to diverse student populations, making it feasible for adoption by other institutions. For student development, for students who struggle with self-regulation, digital literacy training may serve as an accessible and non-stigmatizing pathway to developing self-leadership skills. Unlike interventions that explicitly target “self-regulation” or “self-control,” which may carry negative connotations, digital literacy training is perceived as skill development, potentially reducing resistance and increasing engagement. For addressing the urban–rural gap, given that urban–rural differences have been identified in digital readiness and online self-regulated learning, digital literacy training may serve as a targeted intervention to address regional disparities in students’ initial digital skills and self-regulatory capacities.

### 4.4 Limitations and future directions

Several limitations qualify the conclusions drawn from this study and suggest directions for future research. First, the waitlist control design does not control for nonspecific factors such as attention, expectancy, or social interaction. Participants in the experimental group received 15 h of structured contact time, while the control group received none; thus, it remains unclear whether the observed effects stem from digital literacy content per se or from general engagement and expectancy effects. Future studies should employ an active control group (e.g., general study skills training matched for contact time) to isolate the specific contribution of digital literacy training. Second, all outcomes were assessed via self-report, which may be susceptible to social desirability bias and demand characteristics, particularly in an intervention context where participants may infer the study’s hypotheses. The inclusion of behavioral or objective indicators (e.g., goal attainment logs, digital learning analytics, academic performance records) would strengthen the validity of the findings. Third, the 1-month follow-up provides evidence of short-term maintenance rather than long-term sustainability; although effects remained stable over this interval, extended follow-up assessments (e.g., 6–12 months) are needed to determine whether the intervention produces lasting behavioral change. Fourth, the intervention comprised multiple components (information literacy, digital communication, content creation, security, AI ethics); the present design does not permit disentangling which specific components drive self-leadership improvements. Dismantling studies examining the relative contributions of each module would clarify the active ingredients of the intervention. Fifth, the single-university, single-cultural-context sample limits generalizability. Replication across diverse institutional settings (e.g., vocational colleges, STEM-focused universities) and cultural contexts (e.g., Western individualistic cultures) is essential. Sixth, although the mediation analysis suggests that digital literacy improvement partially accounts for the intervention effect, the simultaneous assessment of changes in digital literacy and self-leadership at T2 precludes definitive conclusions about temporal precedence. It remains possible that self-leadership improvements preceded or co-occurred with digital literacy gains, or that both changes were driven by unmeasured third variables. Finally, the exclusion of two participants based on baseline scores and response patterns, while justified, may introduce minor selection bias; sensitivity analyses confirmed that results remained substantively unchanged when these cases were retained (Group × Time interaction for total self-leadership: *F*(2, 314) = 27.15, *p* < 0.001, *η*^2^ = 0.15, 95% CI [0.08, 0.22]).

## 5 Conclusion

This study contributes to the growing body of evidence that self-leadership is a malleable competency amenable to educational intervention. By demonstrating that digital literacy training enhances self-leadership, the findings bridge two previously separate research strands—self-leadership development and digital education—and suggest that universities can pursue dual-purpose curricula that simultaneously build technical and self-regulatory competencies. The maintenance of effects at the 1-month follow-up provides preliminary evidence of short-term durability; however, longer follow-up periods and behavioral measures are needed to confirm whether such training produces lasting behavioral change. Overall, digital literacy training appears to be a potentially scalable and accessible approach to developing self-leadership, with implications for both curriculum design and student development programs.

## Data Availability

The datasets presented in this study can be found in online repositories. The names of the repository/repositories and accession number(s) can be found in the article/supplementary material.
